# 

**Published:** 1883-04

**Authors:** 


					﻿Whole Somber,
CCCCLIX.
April, 1883.
Number 4,'
Vol. XLVI.
THE
CHICAGO
MEDICAL
Journal & Examiner.
CHICAGO:
PUBLISHED BY THE CHICAGO MEDICAL PRESS ASSOCIATION
188 Clark Street.
[g'Read the Notice of this Journal among Advertisements.
				

